# Behavioral plasticity mitigates the effect of warming on white‐tailed deer

**DOI:** 10.1002/ece3.6087

**Published:** 2020-02-11

**Authors:** Carter L. Wolff, Stephen Demarais, Christopher P. Brooks, Brandon T. Barton

**Affiliations:** ^1^ Department of Biological Sciences Mississippi State University Mississippi State Mississippi; ^2^ Department of Wildlife, Fisheries, and Aquaculture Mississippi State University Mississippi State Mississippi

**Keywords:** behavior, climate change, deer, thermal heterogeneity

## Abstract

Climate change is expected to create novel environments in which extant species cannot persist, therefore leading to the loss of them and their associated ecological functions within the ecosystem. However, animals may employ behavioral mechanisms in response to warming that could allow them to maintain their functional roles in an ecosystem despite changed temperatures. Specifically, animals may shift their activity in space or time to make use of thermal heterogeneity on the landscape. However, few studies consider the role of behavioral plasticity and spatial or temporal heterogeneity in mitigating the effects of climate change. We conducted experiments to evaluate the potential importance of behavior in mediating the net effects of warming on white‐tailed deer (*Odocoileus virginianus*). We used shade structures to manipulate the thermal environment around feeding stations to monitor deer feeding activity and measure total consumption. In individual experiments where deer only had access to unshaded feeders, deer fed less during the day but compensated by increasing feeding during times when temperature was lower. In group experiments where deer had access to both shaded and unshaded feeders, deer often fed during the day but disproportionally preferred the cooler, shaded feeders. Our results suggest that deer can capitalize on temporal and spatial heterogeneity in the thermal environment to meet nutritional and thermal requirements, demonstrating the importance of behavioral plasticity when predicting the net effects of climate change.

## Graphical abstract

Animal behavior may mediate the net effects of climate change within ecosystems but is largely ignored in climate change research. We conducted experiments with shaded feeders to evaluate how temperature alters feeding behavior of white‐tailed deer. Our results show that deer can alter their behavior to take advantage of spatial and temporal heterogeneity in temperature to maintain their functional role as herbivores.

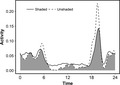

## 1 INTRODUCTION

Anthropogenic climate change is expected to create novel environments that alter the distribution of species and their interactions within ecosystems (Thomas et al., 2004; Walther et al., 2002). Predicting these effects has become a prominent goal in ecology, with countless studies aimed at understanding the fate of species in the Anthropocene (Tylianakis, Didham, Bascompte, & Wardle, 2008; Van Der Putten, Macel, & Visser, 2010). Many approaches, such as predicting range shifts with climate envelopes, have inherently assumed that animals of the future will be constrained by temperature the same way as they are today (Barton, 2011; Schmitz, Post, Burns, & Johnston, 2003). While it is unlikely that most animals will evolve at a rate fast enough to influence the net effects of climate warming (Parmesan, 2006; Quintero & Wiens, 2013), there is a growing appreciation that behavioral plasticity may play an important role (Hoffmann & Sgrò, 2011; Wong & Candolin, 2015). Indeed, animals are unlikely to passively incur the costs associated with climate change. Instead, animals may co‐opt existing traits to mitigate the negative effects of warming and persist in future, novel environments (reviewed in Buchholz et al., 2019). Unfortunately, few climate change studies have explicitly incorporated behavior and our understanding of how animal behavior may influence the net effect of climate change is limited (Harmon & Barton, 2013; Wong & Candolin, 2015).

Animal behavior is likely to influence the net effects of climate change in two ways. First, behavioral plasticity may alter the direct effect of climate change on a species and allow it to maintain its current geographic distribution. For example, organisms in thermally stressful environments may behaviorally thermoregulate by making use of spatial (Bacigalupe, Rezende, Kenagy, & Bozinovic, 2003; Block et al., 2001; Street et al., 2016; van den Berg, Thompson, & Hochuli, 2015) or temporal microclimates (Aublet, Festa‐Bianchet, Bergero, & Bassano, 2009; Carla, Olsen, Knutsen, Albretsen, & Moland, 2016; Hutchison & Maness, 1979; Levy, Dayan, Porter, & Kronfeld‐Schor, 2019). These microclimates may offer thermal refuge within a landscape, which animals can use to remain within suboptimal areas. Second, behavioral thermoregulation may alter inter‐ and intraspecific interactions, thereby generating indirect effects within a community (Blaustein et al., 2010; Cornelissen, 2011; Lensing & Wise, 2006). For example, studies across a broad range of taxa, including both ectothermic and endothermic species, have shown some of the first responses to environmental change are behavioral shifts by consumers (Post, Peterson, Stenseth, & McLaren, 1999; Voigt et al., 2003). Behavioral shifts among consumers can then generate cascading effects on their resources at lower trophic levels (Barton & Schmitz, 2018; Urban, Zarnetske, & Skelly, 2017).

Unfortunately, the role of thermal heterogeneity and its implications for climate change effects are poorly understood (Dobrowski, 2011; Elmore et al., 2017; Sears, Raskin, & Angilletta, 2011). Spatial and temporal thermal heterogeneity may give animals the opportunity to alter their behaviors in ways that mitigate the effects of stressful environments. A large amount of research has focused on how animals move between microenvironments to thermoregulate (Bowyer & Kie, 2009; Carroll, Davis, Elmore, & Fuhlendorf, 2015; Huey & Slatkin, 1976; Kearney, Shine, & Porter, 2009; Long et al., 2014), and evidence suggests that ignoring thermal heterogeneity can result in over‐ or underestimation of the effects of climate warming (Huey, Hertz, & Sinervo, 2003; Sears et al., 2016). The importance of behavior and thermal heterogeneity for understanding the net effects of climate change has been demonstrated in some arthropod systems, where consumers use cooler thermal refuges during hot periods in order to remain within the broader landscape and continue their functional role (Barton & Schmitz, 2009; Harley, 2011). However, it remains unclear how the importance of behavioral plasticity and use of temporal or spatial thermal heterogeneity generalize to other systems, such as endotherms.

To evaluate how animal behavior may mediate the effects of warming in a vertebrate herbivore, we studied temporal and spatial patterns of white‐tailed deer (*Odocoileus virginianus*) feeding behavior in a controlled, replicated experiment. We created thermal heterogeneity within large enclosures by using shaded and unshaded feeding stations and monitored feeding activity 24 hr per day. In large enclosures with multiple deer, we compared the use of shaded and unshaded feeding stations. In smaller enclosures, we presented individuals with either shaded or unshaded feeders to evaluate their behavior and feeding rate. Our approach allowed us to evaluate how deer may alter their behavior to capitalize on spatial (shaded or unshaded feeders) and temporal (day and night) variations in temperature. Specifically, we hypothesize that the presence of shade will allow deer to access feeders more throughout the 24‐hr day, which will lead to greater consumption in shaded feeders relative to unshaded feeders.

## 2 MATERIALS AND METHODS

### 2.1 Study system

White‐tailed deer are a widespread herbivore that is common in North America. At lower latitudes, deer experience high air temperatures in summer months that can elevate core body temperature and create challenging thermal conditions (Demarais, Fuquay, & Jacobson, 1986). Increasing temperatures during the summer may be particularly stressful because this time of year corresponds with lactation, which is energetically expensive (Black, Mullan, Lorschy, & Giles, 1993; Millar, 1977) and can generate heat (Purwanto, Abo, Sakamoto, Furumoto, & Yamamoto, 1990). As dominant herbivores in most of their range, deer fill an important functional role and can affect plant communities by altering plant community composition and growth rate of some species (Russell, Zippin, & Fowler, 2001). Thus, understanding the effects of increasing temperatures on this species is paramount to understanding the net effect of climate change on the ecosystems that they inhabit.

We conducted experiments at the Mississippi State University Rusty Dawkins Memorial Deer Unit (Starkville, MS, USA). The outdoor facility houses wild‐captured deer from Mississippi, as well as some of their captive‐born offspring (Michel, Demarais, Strickland, & Belant, 2015). Experiments were conducted within enclosures of two sizes (described below) and constructed from fencing covered with 70% shade cloth. Enclosure walls, as well as existing hardwood trees, provided some variation in shade available in each enclosure. Moving deer among treatments required anesthetization, which was accomplished using BAM™ (Zoo‐Pharm) delivered with a Pneu‐Dart projection system (Pneu‐Dart, Inc.) and following Mississippi State University approved protocol (IACUC Protocol ID: 17‐491).

### 2.2 Group experiment

**Figure 1:**
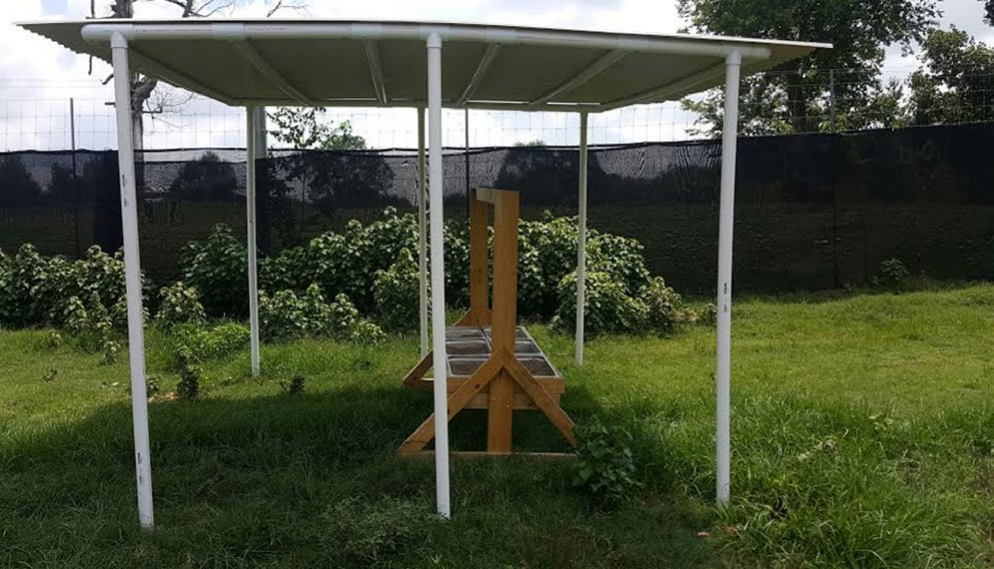
Group experiment feeder and shade canopy. Feeders were constructed with a wooden frame and included six removable feed bins. Two feeders were placed in each large enclosure (~0.75 ha). Over each feeder, we placed a large canopy with either opaque or translucent roofing to simulate shaded or unshaded conditions

We conducted a group experiment using four large enclosures (~0.75 ha each) from April to September 2017. Each enclosure contained two identical wooden feeders (Figure 1) with six metal troughs (0.52 × 0.32 × 0.15 m) per feeder placed in areas exposed to full sun (10 m away from the eastern edge of the enclosure, with feeders 5 m apart). We placed a canopy over each feeder that was constructed from a PVC frame (3.7 m × 3.4 m × 2.2 m) and corrugated roofing material. Each enclosure had both a shaded and unshaded canopy. Shaded canopies were constructed from opaque roofing material while unshaded canopies were constructed from translucent roofing material. The average temperature was 1.4°C warmer under the unshaded feeder relative to the shaded (Wolff unpublished data). We monitored visitation to each feeder using infrared, motion‐triggered camera traps (Bushnell Trophy HD Essential) placed 2.5 m away at a height of 1.5 m. While cameras were triggered via motion, we included a 30‐s delay before the same camera was able to be triggered again.

To initiate the experiment, we added known amounts of commercially available, pelletized feed to the feeders (Cargill Sportsman's Choice Record Rack, Cargill, Inc.). We quantified consumption by returning to the enclosure after 48 hr and weighing the remaining feed. After weighing the remaining feed, we refilled the feeders and weighed them to determine initial feed weight for the ensuing trial. This process was repeated for each trial of the experiment. If strong winds and precipitation led to feed saturated with water, we discarded the feed from the feeder and excluded that data from the analysis. Every 2 weeks, we rotated the opaque and translucent canopy covers to reduce potential bias in treatment location.

The total number of deer per enclosure in the group experiment ranged from 5 to 19 during the study, with approximately a 3:1 ratio of female to male deer for the entire population at the facility. However, we maintained similar deer densities (±1–2 deer between enclosures) and sex ratios within the four enclosures at any point in time. We averaged 48‐hr consumption samples to estimate a daily rate of consumption. To account for differences in density, we adjusted consumption data to a per animal basis based on the number of deer in each enclosure.

We observed camera photographs to quantify feeding behavior at each feeder. We considered a feeding event as any picture that included a standing deer with its head and neck within the perimeter of the feeder canopy. If an individual was captured laying down underneath the canopy, we did not consider that as a feeding event. Thus, our data reflected the frequency of feeder use throughout the day for both the unshaded and shaded feeder. We used the head and neck criterion to standardize the comparison of feeding behavior between shaded and unshaded feeders.

Multiple deer died within one of the enclosures during the experiment and their health status may have affected feed consumption. We excluded data from this enclosure in our analyses to eliminate any potential health‐related biases. Additionally, the presence of nonfocal foraging animals (i.e., opossums and raccoons) occurred infrequently during our experiment. However, we excluded any samples from our analysis that included other foraging animals as their presence would likely bias consumption and the timing of feeding events.

### 2.3 Individual experiment

We conducted feeding trials on individual, female deer in four small enclosures (~0.05–0.07 ha each) from May 2017 to September 2017. In each enclosure, deer were presented with either a shaded or unshaded feeder. Feeders were constructed from a wooden frame (1.2 × 2.4 × 1.5 m), with two metal feeding troughs (0.52 × 0.32 × 0.15 m) per feeder and covered with corrugated roofing material. Each feeder was monitored continuously using an infrared video camera positioned 10 m away from the feeder at a height of 1.8 m. Video cameras were connected to a digital video recorder system (Lorex Technology Inc.).

The experiment was separated into nine 2‐week blocks and each block consisted of four trials. To begin, one deer was randomly assigned to each of the four enclosures. To minimize the effects of the relocation process, deer were placed in the experimental enclosures for 36 hr to acclimate. After 36 hr, we rotated deer counterclockwise to the adjacent enclosure to begin the first trial. During this rotation, we added and recorded initial weight of feed in the feeder (Cargill Sportsman's Choice Record Rack, Cargill, Inc.). We repeated this process for each of the four enclosures. After 72 hr, we rotated deer into adjacent enclosures and repeated the feed processing. At the end of the four‐trial block, deer were removed from the small enclosures and returned to the large enclosures used for the group experiment. Before initiating the next block, one of the two shaded feeders or two unshaded feeders was randomly assigned to the each enclosure. We randomly assigned one feeder to each enclosure to minimize the potential for an enclosure effect on shade treatment. We averaged measurements of consumption across the 72‐hr sampling period to estimate a daily rate of consumption per deer.

We evaluated video data for instances of feeding at each feeder. We considered a feeding event to be when the head and neck of a deer were within the perimeter of the feeder canopy, recording the time each feeding event was initiated. However, if an individual laid down underneath the canopy, we scored that as the end to the feeding event. If the individual got up and its head and neck were still within the perimeter of the canopy, we considered this a new feeding event. Because deer may engage in other, nonfeeding activities while at a feeder (e.g., vigilance, resting), we did not use video to quantify total feeding activity based on duration at the feeder. Additionally, we do not consider the duration of feeding in our original hypotheses. Thus, we used the video data to determine the initiation of feeding events and used the feed weight data to measure consumption.

Similar to the group experiment, we did not frequently encounter other animal foragers. Samples that did show the presence of other animal foragers were again excluded from the analysis.

### 2.4 Statistical analysis

Analyses were completed using the statistical computing language r (R Core Team, 2016). Temporal effects of shade treatments were analyzed using a temporal overlap analysis in the Overlap Package in r (Meredith & Ridout, 2017). We fitted camera and video data to a kernel density and estimated a coefficient of overlap between shaded and unshaded feeders during a 24‐hr day (Biggerstaff, Lashley, Chitwood, Moorman, & Deperno, 2017). In this package and the following CircStats package, time is represented in radians, with the 24‐hr day treated as a circle equivalent to the product of 2π. The coefficient of overlap is defined as

$$\phi \left(s,u\right)=\int \min\left\{s\left(t\right),u\left(t\right)\right\}dt$$

and measures the proportion of time during a 24‐hr day that activity was simultaneously observed under the shaded (s) and unshaded (u) feeders (Ridout & Linkie, 2009). Each function, $s\left(t\right)$ and $u\left(t\right)$ quantifies the density of activity over a day such that $\int s\left(t\right)dt=\int u\left(t\right)dt=1$. The minimum value of $\phi_{\min}=0$ occurs when there is no overlap in the timing of activity because $\min\left\{s\left(t\right),u\left(t\right)\right\}=0$ for all t. Alternatively, the maximum $\phi_{\max}=1$ occurs when the timing of activity at both feeders is equal (even if the activity densities differ). We used bootstrapping with *n* = 1,000 to estimate 95% confidence intervals for the coefficient of overlap.

We used Watson's *U*2 statistic to test for differences in the level of activity at each feeder (sensu Lashley et al., 2018) using the CircStats package in r (Lund & Agostinelli, 2012). Watson's *U*2 test evaluates the hypothesis that diel activity patterns from shaded and unshaded feeders differ.

In addition to comparing activity curves, we fit a negative binomial regression to measure the occurrence of deer at a feeder during each hour of the day. The expected number of deer feeding at a feeder was hypothesized to be a function of ambient, average hourly solar radiation. Solar radiation data for Starkville, MS, from the Soil Climate Analysis Network station (https://www.wcc.nrcs.usda.gov/scan/), and instantaneous measurements of solar radiation were averaged for each hour of each day for the duration of the study. The relationship between visitation to a feeder (y) and solar radiation (x) was modeled using a power function, $y=ax^{b}$, where *a* is a constant, and *b* represents the rate of increase or decrease in visitation rate. We used a maximum likelihood approach in the BBMLE package in r (Bolker & R Development Core Team, 2017) to estimate the parameters *a* and *b* independently for shaded and unshaded models. We then compared the parameters of each model, along with their 95% confidence intervals to determine whether shaded and unshaded models of feeder visitation rate were different. This procedure was conducted separately for the group experiment and the individual experiment.

We analyzed the effects of shade treatments on consumption using general linear mixed effects models in the LME4 package in r (Bates, Maechler, Bolker, & Walker, 2015), assuming a Gamma distribution to account for non‐normal data that were rightly skewed. In the individual experiment, we treated individual deer as a random intercept to control for variation. Within each large enclosure, we collected average daily temperature. We used these temperature recordings to confirm that temperature did not differ significantly among the large enclosures (Wolff unpublished data). However, the presence of hardwood trees and other natural variation may have influenced the abiotic conditions within the large enclosures. To account for this, we treated the enclosure as a random intercept. In addition to using shade treatment as a binary predictor (present, absent), we also included three different models with daily minimum, daily maximum, and daily average temperature. Daily temperature data for Starkville, MS, was accessed from the Soil Climate Analysis Network station (https://www.wcc.nrcs.usda.gov/scan/) and averaged across the 48‐ or 72‐hr sampling period for the group and individual experiments, respectively. We compared models from each experiment using AIC values, from the MuMIn package in r (Bartoń, 2018).

## 3 RESULTS

### 3.1 Group experiment

**Figure 2:**
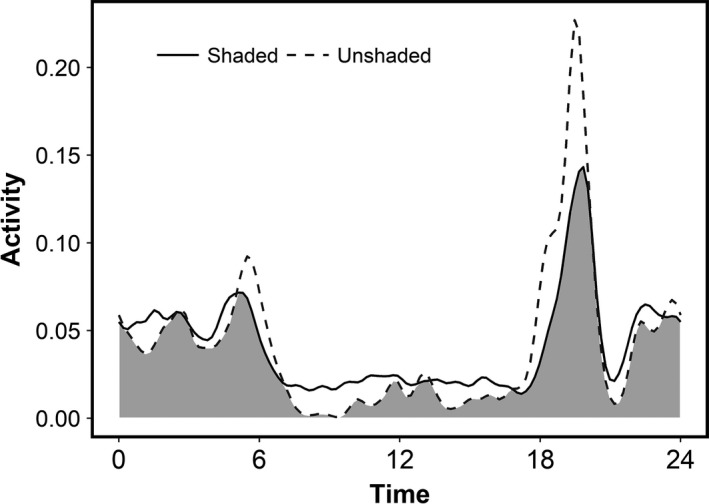
Overlap plot of activity curves in the group experiment. Feeding activity patterns of deer were different between shaded (solid black line) and unshaded (dashed gray line) feeders (coefficient of overlap = 0.825; Watson's *U*2 statistic, *p* < .001). The gray area represents areas of overlap between the two activity patterns

Activity patterns of the group experiment resulted in a coefficient of overlap of 0.825 (bootstrap 95% CI 0.819–0.831; Figure 2). Further analysis of these activity patterns indicated that shaded and unshaded feeders were used differently throughout the 24‐hr day (Watson's *U*2 test, *p* < .001). This was most evident during the mid‐day where feeding activity at the shaded feeder was proportionally greater compared with the unshaded feeder and crepuscular periods where feeding activity at the unshaded feeder was proportionally greater compared with the shaded feeder.

Estimates for feeder visitation rate as a function of hourly solar radiation did not yield statistically different models for shaded and unshaded feeders. In shaded feeders, parameters *a* and *b* were estimated to be 35.723 (95% CI: 21.362–50.084) and 0.080 (95% CI: −0.043 to 0.204), respectively. In unshaded feeders, parameters *a* and *b* were estimated to be 26.954 (95% CI: 16.163–37.745) and 0.099 (95% CI: −0.019 to 0.217), respectively.

**Figure 3:**
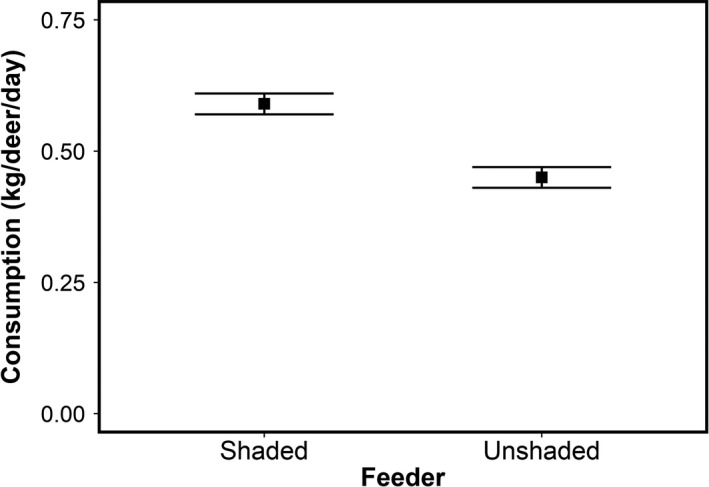
Consumption in the group experiment. Consumption differed among shaded and unshaded feeder treatments. Deer consumed 23% less in unshaded feeders (Wald test, *p* = .0494). Shade treatment and average temperature across the 2‐day sampling period influenced daily consumption. Daily consumption rates were averaged across the sampling period and by the total number of deer per trial. Each point is the mean daily consumption. Error bars are ±one standard error

In the analysis of consumption at shaded or unshaded feeders, the model using average temperature had a lower AICc value than models using either minimum or maximum temperature. However, the AICc values did not differ when comparing the model using average temperature to the model that only used shade treatment (Table 1). Consumption at shaded feeders was 0.59 ± 0.02 (mean ± 1 *SE*) kg per deer per day while consumption at unshaded feeders was 0.45 ± 0.02 (mean ± 1 *SE*) kg per deer per day (Figure 3). Daily per‐deer consumption rate was 23% lower at unshaded feeders in the model with shade treatment and average temperature (Wald test, *p* = .0494). Neither average temperature nor the interactive effect of shade treatment and average temperature was significant.

**Table 1:** Model selection for consumption analysis in the group experiment

Model	*df*	Log likelihood	AICc	ΔAIC
Feeder	4	−122.225	252.5	0.00
Feeder * Average Temperature	6	−120.638	253.5	0.93
Feeder * Maximum Temperature	6	−120.800	253.8	1.26
Feeder * Minimum Temperature	6	−121.824	255.9	3.31

Note

We used AICc values to compare models including the shade treatment at the feeder and different measurements of temperature as an assessment of thermal comfort.

### 3.2 Individual experiment

**Figure 4:**
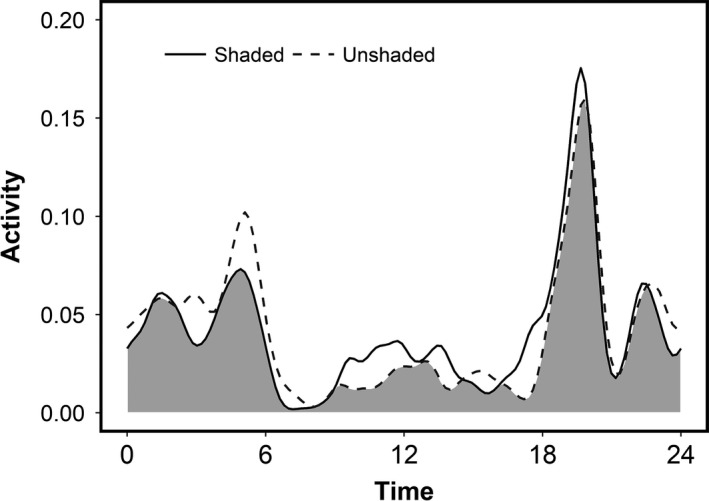
Overlap plot of feeding activity curves in the individual experiment. Feeding activity patterns of individual deer were different between shaded (solid black line) and unshaded (dashed gray line) feeders (coefficient of overlap = 0.867; Watson's *U*2 statistic, *p* < .001). The gray area represents areas of overlap between the two activity patterns

Activity patterns between the shaded and unshaded feeders resulted in a coefficient of overlap of 0.868 (bootstrap 95% CI 0.837–0.874; Figure 4). As with the group experiment, deer used shaded and unshaded feeders differently across the 24‐hr day (Watson's *U*2 test, *p* < .001). Again, this was most evident during mid‐day when feeding activity at the shaded feeder was proportionally greater and during crepuscular periods when feeding activity at the unshaded feeder was proportionally greater.

Fitting solar radiation observations of visitation rate to shaded feeders resulted in estimations for parameters *a* and *b* to be 34.855 (95% CI: 20.926–48.785) and 0.123 (95% CI: 0.007–0.240), respectively. In unshaded feeders, parameters *a* and *b* were estimated to be 32.789 (95% CI: 19.634–45.945) and 0.077 (95% CI: −0.045 to 0.199). Overlapping confidence intervals for each parameter between shaded and unshaded feeders suggests that average hourly solar radiation does not influence visitation to a feeder.

**Figure 5:**
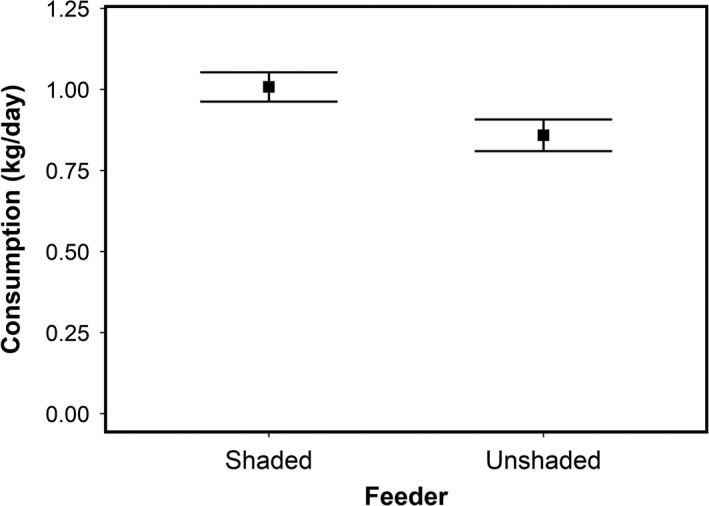
Consumption in the individual experiment. Consumption differed among shaded and unshaded feeder treatments. Deer consumed 17% less food in unshaded feeders (Wald test, *p* = .0169). Measurements were taken across a 3‐day sample period and averaged to estimate a daily rate of consumption. Each point is the mean daily consumption. Error bars are ±one standard error

Models that included temperature had higher AICc values than the model with shade treatment only (Table 2). Deer consumed 1.00 ± 0.04 (mean ± 1 *SE*) kg of feed per day at shaded feeders and 0.85 ± 0.05 (mean ± 1 *SE*) kg of feed per day at unshaded feeders (Figure 5), suggesting that deer ate 17% less feed per day when feeders were unshaded (Wald test, *p* = .0169).

**Table 2:** Model selection for consumption analysis in the individual experiment

Model	*df*	Log likelihood	AICc	ΔAIC
Feeder	4	−42.453	93.2	0.00
Feeder * Minimum Temperature	6	−40.954	94.6	1.36
Feeder * Maximum Temperature	6	−42.262	97.2	3.98
Feeder * Average Temperature	6	−42.416	97.5	4.28

Note

We used AICc values to compare models including the shade treatment at the feeder and different measurements of temperature as an assessment of thermal comfort.

## 4 DISCUSSION

We show that deer altered their behavior to meet conflicting thermal and nutritional demands in ways that may allow them to maintain their functional role as herbivores as climate warms. Consistent with previous work (e.g., Ager, Johnson, Kern, & Kie, 2003; Carranza, de Trucios, Medina, Valencia, & Delgado, 1991; Relyea & Demarais, 1994), the deer in our experiments were largely crepuscular. While deer in all treatments exhibited some daytime feeding, this behavior was more common when feeders were shaded. In other words, when presented with the opportunity to feed during the day without exposure to direct sunlight, deer took advantage of the shaded feeder and fed during the daytime. In contrast, when feeders were unshaded and deer were forced to chose between avoiding thermal stress or foraging, deer largely forfeited daytime feeding. However, deer partly compensated by increasing feeding activity during the crepuscular periods (Figure 4). Thus, deer demonstrated the ability to capitalize on temporal variation in temperature to meet nutritional demands despite more extreme environmental conditions.

Similarly, the group experiment demonstrated that deer may be able to capitalize on spatial variation in temperature to mitigate the effects of warming. Deer fed from shaded and unshaded feeders at similar levels during the night when the two shade treatments did not differ in temperature. However, deer showed a strong preference for the shaded feeder during the daytime and dramatically reduced their use of the unshaded feeder (Figure 2). Thus, deer were able to capitalize on differences in microhabitats to forage without exposing themselves to harsh environmental conditions. The important message to learn from spatial and temporal shifts in feeding behavior is that these herbivores did not behave the same way in the two different environments. Instead, deer exhibited behavioral plasticity, using their environment in a different way in response to the changed thermal landscape. Although this result is not suprising, most climate change predictions have the underlying assumption that extant animals are identical to future animals. In doing so, studies inherently ignore the importance of context dependence in behaviors that may mediate the net effects of climate change.

Although our study shows that deer may be able to alter their feeding behavior in ways that mitigate the effects of warming, there may still be consequences for deer and their interactions within communities. The individual experiment revealed a 17% reduction in average daily consumption when deer were restricted to unshaded feeders. While this level of decreased food intake may not be detrimental during short periods of time (McCarter, Masoro, & Yu, 1985), long‐term reduction in food intake could have consequences, such as reduction in body size and fecundity, that may impact species at both the individual and population levels (Milner, Van Beest, Schmidt, Brook, & Storaas, 2014; White, 1983). This may be especially influential during the hot summer months that correspond to lactation, which is a substantial energetic cost for female deer (Black et al., 1993; Millar, 1977; Purwanto et al., 1990). Although it is unclear how deer would respond if exposed to unshaded feeders for longer time periods, our results reveal the potential for increasing temperatures to impact deer nutrition by altering the amount of food they consume.

Altered deer consumption may indirectly affect other species. In the group experiment, deer consumed 23% less from unshaded feeders than shaded feeders (Figure 3). This suggests that heterogeneity in the thermal environment can lead to spatial heterogeneity in herbivory. Even if deer were able to consume the same amount of food at a landscape level, a shift in where they feed could have top‐down effects on plants at smaller spatial scales (Cahoon, Sullivan, Post, & Welker, 2012; Tylianakis et al., 2008). For example, increasing temperatures may concentrate deer activity into isolated, cooler microhabitats analogous to our shaded feeders, while simultaneously releasing plants in warmer microhabitats from herbivory. Concentrating deer activity in cooler microhabitats may also increase their encounter rates with other species, such as predators. While our study did not include predators, it is possible that predation risk may further influence how deer balance nutritional and thermal demands (Lowrey et al., 2019). This may be especially important for deer as recent work has shown shifts to a diurnal pattern in the presence of predators (Crawford et al., 2019; Higdon, Diggins, Cherry, & Ford, 2019). Making use of thermal heterogeneity in a habitat could impact this diurnal shift. Evaluating these potential top‐down and bottom‐up effects that arise from altered deer behavior is beyond the scope of this study, but a laudable next step in understanding the net effects of climate change on deer and their ecosystems.

As with any manipulative experiment, the price of control and replication is a reduction in realism. Using shade as a proxy for temperature may have confounded the effect of temperature and light on foraging behavior of deer. We did not measure incoming wavelengths of light at each feeder and thus are limited by this autocorrelation between light and temperature. While we attempted to reduce variation in this aspect, our study is limited by using shade to create two different thermal environments. In addition, confining deer to areas much smaller than their natural home ranges may introduce variation or experimental artifacts. Movement patterns in ungulates are commonly driven by forage availability, and restricting this movement may lead to an under‐ or overestimation of the natural activity patterns of our deer (Frair et al., 2005). Furthermore, our study used pelletized feed to allow systematic measurement of consumption rates. The feed is more nutritious than many plants available to wild deer, and it is unclear how the nutritional quality of food may alter the outcomes of consumption and time spent foraging (Parker, Barboza, & Gillingham, 2009; Wilmshurst, Fryxell, & Bergman, 2000). Social behavior of a species, such as deer, could have differentially influenced the results between individual and group feeding experiments. Previous research has suggested that group size may increase the time spent feeding by deer (Lashley et al., 2014), meaning that altering group size throughout the experiment may bias our estimation of consumption and individuals might not behave the same as groups. We reduced this bias by implementing a paired design within enclosures and by maintaining similar densities of deer in each enclosure, but it is still important to consider. Furthermore, our feeders limited the number of deer that could feed simultaneously, which differs from natural foraging where animals could be spaced across a larger patch. Deer may have been discouraged from using an already crowded feeder, especially if individuals exhibited dominance behavior to other individuals within the group (Stone, Cherry, Martin, Cohen, & Miller, 2017). Ultimately, this could bias our assessments of feeder use if individuals were using a suboptimal feeder due to lower social rank. However, this effect is weakened when food is abundant (Michel, Demarais, Strickland, Belant, & Millspaugh, 2016), as it was in our experiment.

## 5 CONCLUSION

Our results suggest that deer can alter their behavior in ways that may mitigate the negative effects of a warming climate. These animals made use of spatial and temporal heterogeneity in temperatures in ways that may allow them to maintain their function as herbivores as temperatures increase. Thus, our results corroborate recent literature that argues for the incorporation of animal behavior into climate change studies (Abernathy et al., 2019; Buchholz et al., 2019; Harmon & Barton, 2013; Wong & Candolin, 2015). Further, our results demonstrate that managing habitats to provide thermal heterogeneity may allow wildlife to alter their behavior in ways that may ameliorate the effects of climate change.

## AUTHOR CONTRIBUTION

BTB and SD conceived and designed the study, with the input from all authors. CLW conducted the experiment and collected data, which he analyzed with significant help from CPB. CLW wrote the manuscript, with feedback and guidance provided by BTB, SD, and CPB.

## Data Availability

Data collected and used in this were available on figshare at the url https://doi.org/10.6084/m9.figshare.11444574.
